# Dentists Slandering Each Other

**Published:** 1853-10

**Authors:** 


*Dentists Slandering each other.*—We scarcely know how to reply to our fair correspondent, whose letter, under the head of professional etiquette, we published in another part of the present No. of the Journal. She has been unfortunate, it must be confessed, in her intercourse with dentists, and we would be hard hearted indeed, if we did not sympathise with her in her distress. The prospect of losing two central incisors, is enough to fill the heart of any beautiful young lady with sorrow. But we trust she has had the good fortuneere this, to secure the services of some one more worthy of confidence than Drs. 2, 3, and 4, and the other gentlemen of the profession on whom she called. If so, she has realized that the "little blue bright blades" with their "jeweled handles," in skillful, experienced hands, are not the terrible things she had pictured them to be in her imagination.

The spirit of illiberality of which our fair friend speaks, to call it by no harsher name, is not so universal among dentists as she seems to suppose. That it exists at all, is, certainly, very much to be regretted. That any member of the dental profession should be so lost to shame, and so destitute of gentlmanly feeling, as well as of a proper sense of self-respect, as to descend to the use of such unworthy and reprehensible means to obtain practice, is a source of sorrow to all honorable, high minded practitioners. It may sometimes succeed with ignorant unsuspecting individuals, but the selfish motive is too apparent not to be seen through by most persons, and whether it has its origin in jealousy or love of gain, it is equally despicable.

We are not surprised, therefore, that one possessed of such fine feelings, and so just an appreciation of common courtesy as our fair correspondent, should, from the disrespectful manner in which the dentists on whom she called, spoke of each other, have formed an unfavorable opinion of the whole profession. If a dentist thinks to elevate himself in public estimation by traducing his neighbor, the letter of our fair friend will show to him how sad a mistake he has committed, and we would that it could be read by every one who resorts to such dishonorable means to obtain practice. The rebuke, not more severe than just, will, we hope, be productive of good, and we publish the letter for the benefit of those interlopers of the profession—pseudo-practitioners, for we do not believe that a well educated dentist would stoop to such meanness—whose chief claim to consideration seems to consist in unscrupulous self-laudation and a readiness to disparage the merits of others.

So much for the *tares* of the profession, which, we are sorry to confess, have sprung up rather thickly in our midst, but we are consoled by the belief, that time and good culture will ultimately weed them out. The number of well educated dentists is rapidly increaing. Even now, almost every town and city in the union, can boast of one or more practitioners of this description.

We have now answered, to the best of our ability, the questions, (and they have perplexed us not a little,) proposed by our fair friend, Kate, and if the explanation we have been able to give, shall have the effect of removing from her mind the unfavorable impression which she seems to have formed of our profession, for we would not on any account have her continue in so unjust a belief, we shall not regret having attempted to comply with her wishes. Having said thus much, we will promise, but mind, in doing so, we are not influenced by any interested motive, should we ever have the pleasure of meeting her in our *drawing* room, to do all in our power to divest the "keen paraphernalia of our private inquisition," of the terrors with which they seem to have inspired her sensitive nature.

We scarcely know whether to regard the playful hit which she makes at practitioners of the other departments of medicine as intended to be personal or not, having in years long since gone-by exercised the vocation of a physician, but be that as it may, it is so prettily made, we cannot find it in our heart to take exception to this part of her letter. Our friend, Dr. Piggot, however, it being a matter in which he, at this time, is more immediately interested, may feel a little sensitive upon the subject, but, in this instance, we shall exercise the prerogative of our editorial seniority, and insist upon his bearing it without a word of reply.

